# Supplementary material for: Surface Microbiome Profiling of Dental Elevators Using Third‐Generation Sequencing: Implications for Infection Control in Dental Practice
Source: Microbiologyopen. 2025 Dec 8;14(6):e70178. doi: 10.1002/mbo3.70178 (PMC12685768; doi:10.1002/mbo3.70178)
Supplement: Supplementary file 1 — customer_backup. [file MBO3-14-e70178-s001.zip › customer_backup/customer_backup/taxa_summary/krona/samples/M06.Krona.html]

Javascript must be enabled to view this page.

members
magnitude
magnitudeUnassigned

M06.krona

53197

53190

1

1

1

1

1

1

4264

1

1

1

1

1

3145

3145

50

12

12

1

1

8

8

3

3

20

20

2

2

4

4

3095

262

228

34

2833

276

2296

251

10

9

6

4

4

4

2

2

2

3

2

2

1

1

1

1

1

12

6

6

6

6

2

2

2

2

4

2

2

1

1

2

2

2

1097

179

179

4

4

175

1

19

1

6

55

11

67

15

168

167

20

20

123

123

4

4

20

20

1

1

1

17

17

17

17

4

4

4

1

1

2

667

667

580

580

68

1

46

7

3

11

19

19

62

53

1

1

48

48

1

1

1

1

2

1

1

1

1

1

7

5

5

1

1

1

1

1

1

1

1597

1597

1597

1597

1597

322

1

98

330

36

1

291

208

310

16

16

16

16

16

16

3937

3937

3937

3937

3937

192

3728

17

9865

9865

9865

9619

10

10

9609

8356

1253

246

246

32

38

57

13

10

17

23

56

1

1

1

1

1

1

2

1

1

1

1

1

1

1

1

1

1

67

3

3

3

1

1

2

2

64

64

64

64

64

1

1

1

1

1

1

3

1

1

1

1

1

1

1

1

1

1

1

1

1

1

1

600

4

4

4

4

4

596

596

371

48

48

32

32

233

233

58

58

224

224

224

1

1

1

657

657

657

657

598

217

281

100

59

59

13

13

1

1

1

1

12

1

1

1

10

10

10

1

1

1

1

1

1

1

1

1

1490

128

59

59

59

59

1

1

1

1

56

4

1

1

3

2

1

40

8

8

8

1

7

19

19

5

3

2

3

1

1

2

2

1

1

1

8

2

2

1

1

1

1

4

4

1

1

1

1

1

1

1

1

1

1

1

1

8

8

1

1

1

1

6

3

2

1

1

1

1

1

1362

990

6

6

6

18

6

6

12

12

26

19

19

6

6

1

1

408

407

407

1

1

532

241

241

42

7

35

208

208

41

41

114

1

1

1

9

4

4

4

4

1

1

99

5

5

31

8

23

63

63

2

2

2

3

3

3

1

1

1

1

9

9

9

3

6

2

2

2

2

199

199

199

130

69

1

1

1

1

1

1

1

1

6

1

1

1

5

3

3

2

1

1

1

1

1

1

38

24

24

23

1

14

14

14

5

5

5

5

1

1

4

4

21249

10540

591

591

591

591

156

156

140

140

16

16

32

32

32

32

2641

2269

5

5

3

3

5

1

4

18

18

64

64

485

457

2

26

71

10

61

30

30

40

33

7

8

1

2

5

20

9

11

129

129

9

9

26

26

43

43

53

53

55

55

64

62

2

3

2

1

92

92

5

5

7

7

75

12

63

61

61

1

1

112

6

106

2

2

400

1

76

2

239

25

55

2

366

232

134

3

3

14

14

372

372

372

6685

1552

164

164

186

12

174

524

454

70

1

1

490

490

160

154

6

27

27

3830

1

1

2107

2107

64

64

1635

1635

23

22

1

1303

1303

1303

5

5

5

3

2

7

5

5

5

1

1

1

1

1

1

379

318

9

1

6

1

1

108

108

8

1

7

1

1

2

2

190

188

2

48

48

48

6

1

1

4

4

1

1

7

7

7

44

18

18

18

26

26

26

6548

10

10

10

1

1

8

191

4

4

3

1

187

187

187

6

1

1

1

5

5

5

1

1

1

1

4613

1

1

1

14

14

14

3352

3346

110

1993

8

6

17

1210

2

6

6

3

3

1

2

1242

2

2

1

1

5

4

1

9

6

3

208

170

4

34

11

11

586

19

562

5

7

7

405

382

23

2

2

3

3

3

1

2

1

1

1

29

23

23

18

1

2

1

1

6

4

4

1

1

1

1

282

282

282

50

54

178

14

14

14

14

1402

771

44

44

2

1

1

723

723

2

2

631

32

32

3

3

221

221

85

85

1

1

1

1

18

18

2

2

80

80

188

188

4161

4161

1528

1

1

701

701

826

166

103

69

25

1

162

31

34

235

2632

118

93

25

1019

1019

1428

1

497

775

155

67

62

5

1

1

1

8

8

8

8

8

8

17

17

17

17

17

17

9396

9396

1

1

1

1

755

744

744

2

50

636

56

6

6

6

5

5

5

8475

12

12

12

5

5

5

7

7

7

75

2

2

28

28

45

45

34

33

2

31

1

1

1005

1005

1005

1631

1631

610

990

31

9

9

1

2

1

1

4

652

651

596

55

1

1

237

237

237

4808

519

56

16

3

444

2

2

2150

18

80

451

85

185

6

10

39

60

177

17

1006

12

4

2137

52

56

209

408

208

351

62

25

190

576

163

163

163

24

139

2

2

1

1

1

1

2

2

2

2

2

2

2

5

1

1

1

1

1

1

3

3

3

3

3

3

1

1

1

1

1

1
